# Supplementary material for: Internal structure of the action and acceptance questionnaire II (AAQ-II): evidence for a three-factor and bifactor model in two samples of university students
Source: PeerJ. 2025 Jul 9;13:e19620. doi: 10.7717/peerj.19620 (PMC12255244; doi:10.7717/peerj.19620)
Supplement: Supplemental Information 1 — PM, Painful memories; EFD, emotional/functional distress; AC, affective control; *, Spanish translation. These scores were used for statistical analysis to describe the psychometric evidence for the AAQ-II in university students. [file peerj-13-19620-s001.docx]

**Supplementary material**

**Table S1***Items descriptions and Spanish translation of the Action and Acceptance Questionnaire II*

|  |  |  | Factor |
| --- | --- | --- | --- |
| Item 1 | My painful experiences and memories make it difficult for me to live a life that I would value |  | PM |
|  | *Mis experiencias y recuerdos dolorosos hacen que me sea difícil vivir la vida que querría** |  |  |
| Item 2 | I'm afraid of my feelings |  | ED |
|  | *Tengo miedo de mis sentimientos** |  |  |
| Item 3 | I worry about not being able to control my worries and feelings |  | ED |
|  | *Me preocupa no ser capaz de controlar mis preocupaciones y sentimientos** |  |  |
| Item 4 | My painful memories prevent me from having a fulfilling life |  | PM |
|  | *Mis recuerdos dolorosos me impiden llevar una vida plena** |  |  |
| Item 5 | Emotions cause problems in my life |  | AC |
|  | *Mis emociones interfieren en cómo me gustaría que fuera mi vida** |  |  |
| Item 6 | It seems like most people are handling their lives better than I am |  | AC |
|  | *Parece que la mayoría de la gente lleva su vida mejor que yo** |  |  |
| Item 7 | Worries get in the way of my success |  | AC |
|  | *Mis preocupaciones interfieren en el camino de lo que quiero conseguir** |  |  |
| PM=Painful memories; EFD=Emotional distress; AC=Affective control; *=Spanish translation. | | | |
