# Supplementary material for: Internal structure of the action and acceptance questionnaire II (AAQ-II): evidence for a three-factor and bifactor model in two samples of university students
Source: PeerJ. 2025 Jul 9;13:e19620. doi: 10.7717/peerj.19620 (PMC12255244; doi:10.7717/peerj.19620)
Supplement: Supplemental Information 2 — a, Three-factor model found in the current study; b, Three-factor model found by Pennato et al. (2022) and Arias, Barraca & García (2023). c, Three-factor model found by Spencer et al. (2022). RMSEA, Root mean square error of approximation; SRMR, Standardized root mean square; CFI, Comparative fit index; TLI, Tucker-Lewis index; GFI, Goodness of fit index; AIC, Akaike information criteria; BIC, Bayesian information criteria. These scores were used for statistical analysis to describe the psychometric evidence for the AAQ-II in university students. [file peerj-13-19620-s002.docx]

**Supplementary material**

**Table S2***Complementary CFA results*

|  | Absolute fit | | | | |  | Relative fit | | |  | Parsimony | |
| --- | --- | --- | --- | --- | --- | --- | --- | --- | --- | --- | --- | --- |
|  | χ² | df | χ²/df | RMSEA | SRMR |  | CFI | TLI | GFI |  | AIC | BIC |
| Three-factor_a_ | 97.350 | 11 | 8.850 | .097 | .019 |  | .980 | .961 | .987 |  | 19390.632 | 19503.947 |
| Single-factor | 472.335 | 14 | 33.738 | .199 | .046 |  | .892 | .839 | .944 |  | 19760.069 | 19792.531 |
| Three-factor_b_ | 150.697 | 11 | 13.700 | .124 | .025 |  | .967 | .937 | .982 |  | 19444.044 | 19557.358 |
| Three-factor_c_ | 172.830 | 12 | 14.403 | .129 | .030 |  | .962 | .934 | .944 |  | 4291.814 | 4324.839 |

a=Three-factor model found in the current study; b=Three-factor model found by Pennato et al. (2022) and Arias et al. (2023). c=Three-factor model found by Spencer et al. (2022). RMSEA=Root mean square error of approximation; SRMR=Standardized root mean square; CFI=Comparative fit index; TLI=Tucker-Lewis index; GFI=Goodness of fit index; AIC=Akaike information criteria; BIC=Bayesian information criteria.
