# Supplementary material for: Internal structure of the action and acceptance questionnaire II (AAQ-II): evidence for a three-factor and bifactor model in two samples of university students
Source: PeerJ. 2025 Jul 9;13:e19620. doi: 10.7717/peerj.19620 (PMC12255244; doi:10.7717/peerj.19620)
Supplement: Supplemental Information 4 [file peerj-13-19620-s004.docx]

*Codebook for variables in the dataset*

| Variable | Description | Code |
| --- | --- | --- |
| sex | Sex. | 1=Male; 2=Female. |
|  |  |  |
| item01 | AAQ-II item 01 “My painful experiences and memories make it difficult for me to live a life that I would value” | 1=Never true; 2=Very seldom true; 3=Seldom true; 4=Sometimes true; 5=Frequently true; 6=Almost always true; 7=Always true |
| item02 | AAQ-II item 02 “I’m afraid of my feelings” |  |
| item03 | AAQ-II item 03 “I worry about not being able to control my worries and feelings” |  |
| item04 | AAQ-II item 04 “My painful memories prevent me from having a fulfilling life” |  |
| item05 | AAQ-II item 05 “Emotions cause problems in my life” |  |
| item06 | AAQ-II item 06 “It seems like most people are handling their lives better than I am” |  |
| item07 | AAQ-II item 07 “Worries get in the way of my success” |  |
|  |  |  |
